# Curcumin in High Doses Reverses the UV-B-Induced *DNMT* and *HDAC* Upregulation In Vitro: A Novel Anti-Cancer Approach?

**DOI:** 10.3390/ph19030496

**Published:** 2026-03-17

**Authors:** Afshin Zand, Bence L. Raposa, Dávid Szép, John M. Macharia, Ghodratollah Nowrasteh, Ferenc Budán, Tímea Varjas

**Affiliations:** 1 Department of Public Health Medicine, Medical School, University of Pécs, 7621 Pécs, Hungary; afshin.zand@aok.pte.hu (A.Z.);; 2 Institute of Basics of Health Sciences, Midwifery and Health Visiting, Faculty of Health Sciences, University of Pécs, 7621 Pécs, Hungary; 3 Institute of Physiology, Medical School, University of Pécs, 7621 Pécs, Hungary; szep.david@pte.hu; 4 Doctoral School of Health Sciences, Faculty of Health Sciences, University of Pécs, 7621 Pécs, Hungary

**Keywords:** curcumin, UV radiation, DNMT, HDAC, chemoprevention

## Abstract

**Background/Objectives:** The primary mechanisms driving UV-induced carcinogenesis include DNA damage leading to mutations, and reactive oxygen species (ROS) formation that can cause inflammation, immunosuppression, alteration of the structure of proteins, including transcription factors, and carcinogenesis through epigenetic modifications. Curcumin has the potential to inhibit DNA-methyltransferases (DNMTs) and histone deacetylases (HDACs), but this has not been examined yet at the gene-expression level. In this article, we aimed to explore the potential protective effect of curcumin against UV radiation-induced *DNMT1*, *DNMT3A*, *DNMT3B*, *HDAC5*, and *HDAC6* expression in immortalized keratinocytes (HaCaT), hepatocellular carcinoma (HepG2), and lung adenocarcinoma (A549) cells. **Methods:** Cells were exposed to UV-B radiation for different periods and treated with curcumin at different concentrations to evaluate dose-related trends in DNMT and HDAC gene expression compared with untreated UV-exposed cells. **Results:** UV exposure increased the DNMT and HDAC gene expression levels in the examined cells dose-dependently. Curcumin exposure resulted in decreased mRNA expression levels of DNMT and HDAC gene expression. In our experimental setup curcumin modulated the transcription of DNMT and HDAC genes in A549 and HaCaT cells in a dose-dependent manner. In HepG2 cells, UV-B induced a less pronounced, but still significant, increase in the examined gene expression levels. This effect was also dose-dependently decreased by curcumin, although less markedly. **Conclusions:** Future studies are warranted to examine if curcumin combined with other chemopreventive agents through the HDAC and DNMT inhibitory activity at the gene expression level can exert a synergistic effect and may potentially supplement cancer therapeutic strategies.

## 1. Introduction

### 1.1. Global Burden of Cancer

With 9.7 million cancer-related deaths reported globally in 2022, cancer ranks as the second leading cause of death after cardiovascular diseases [1]. The worldwide economic impact of cancer between 2020 and 2050 is estimated to reach $25.2 trillion (adjusted to 2017 constant prices), accounting for approximately 0.55% of the global gross domestic product [2]. In 2019, cancer was responsible for an estimated 250 million disability-adjusted life years (DALYs), marking a 16.0% increase since 2010 [3].

### 1.2. Epigenetic Factors of Carcinogens and Anticancer Effects

Epigenetics refers to heritable changes in gene expression that occur without changes in DNA sequence. The epigenetic modifications involve changes in components of chromatin—DNA, RNA, and proteins like histones—through specific enzyme-driven covalent bonds [4]. These changes are influenced by factors such as mutations, aging, nutrition, and the environment [5]. DNA methylation and histone acetylation changes can lead to an altered balance between active and inactive chromatin structures, ultimately resulting in modified transcriptional activity [6,7].

The aberrant alteration of epigenetic regulation of cell homeostasis is the key mechanism of the progression of the malignantly transformed cell. Evidence supports that environmental carcinogens not only may cause mutations but also, apart from that, alter the epigenetic patterns in a malignant way [8]. On the other hand, chemopreventive compounds have the potential to impact different aspects of the epigenetic machinery and restore regular gene activity [9]. Hence, polyphenols with anticancer properties can restore the chemical carcinogen-induced elevation of DNA methyltransferase (*DNMT*) and histone deacetylase (*HDAC*) genes [10]. Thus, the ability to ameliorate harmful cellular effects by chemopreventive agents is also reflected in cells’ epigenetic properties; for example, LINE-1 retrotransposon hypomethylation and global DNA hypomethylation are relevant biomarkers of genetic instability, indicating malignant transformation or inflammation [11].

Among post-translational histone modifications, the most important is histone acetylation, which alters the chromatin structure and influences DNA transcription factors, contributing to epigenetic regulation of gene expression [12]. Furthermore, histone modifications are linked to DNA methylation, as DNMTs induce HDACs, leading to histone deacetylation followed by transcriptional repression of tumor-suppressor genes that are typically silenced during oncogenic transformation, ultimately inhibiting differentiation, cell cycle arrest, and apoptosis [12].

DNA methylation is also one of cancer’s most extensively studied epigenetic modifications [13]. Since methylation level varies throughout the cell cycle, 5-methylcytosine (5mC) is often referred to as the “dynamic fifth base” of the genetic code [14]. DNMT enzymes generate methylation at the 5 position of cytosine (C5-methylation), a critical epigenetic mechanism that regulates gene expression [15]. The DNMT enzyme family consists of four active members, with DNMT1 playing a critical role in regulating methylation, while DNMT3 is responsible for de novo methylation [16,17]. Upregulation of DNMT expression is associated with poor prognosis for lung cancer [18], hepatocellular carcinoma [19], and melanoma [20]. Furthermore, UVB-induced skin tumor progression has been linked to altered DNA methylation patterns mediated by methylation-regulating enzymes in in vivo models [21].

Histone acetyltransferase (*HAT*) enzymes and *HDAC* enzymes carry out lysine acetylation or deacetylation on histone proteins. They play essential roles in the epigenetic regulation of gene expression by determining the acetylation status of histone proteins, which determines whether chromatin has a “condensed” structure in the case of heterochromatin or a “relaxed” structure in the case of euchromatin. If histones are acetylated, it results in an euchromatin structure, and the correlating gene expression is increased. The main enzymes responsible for regulating the structure of chromatin are the HDAC enzyme families, and they can be mutated in somatic or germ cells due to exposure to carcinogens or the aging process [22]. HDACs, which are often overexpressed in various tumors, for example, melanoma, are categorized into four classes (I, II, III, and IV) and include 18 different types (HDAC1-11 and SIRT1-7) [23].

### 1.3. The Effects of UV Light

Ultraviolet (UV) light, especially the UV-B range (280–320 nm), is an environmental risk factor associated with the development of skin cancers, including basal cell carcinoma, malignant melanoma, squamous cell carcinoma, and Merkel cell carcinoma [24].

The primary mechanisms driving UV-induced carcinogenesis include DNA damage leading to mutations, and reactive oxygen species (ROS) formation that can cause inflammation, immunosuppression, and carcinogenesis through epigenetic modifications [25]. UV irradiation directly causes DNA damage and inactivates P53 [26,27,28]. DNA methylation increases the likelihood of UV-induced DNA damage [27]. UV light can affect the expression of various genes, including oncogenes, tumor suppressor genes, growth factors, cytokines, and their receptors. For example, UV radiation can inactivate P53 through mutation, thereby inducing *DNMT* and *HDAC* expression and consequently increasing DNA methylation activity, silencing tumor suppressor genes, and forming a positive feedback loop [28]. That can influence intracellular signaling, leading to a divergence of increased cell proliferation rate or activating tumor suppressor effects [29].

Not only UV directly, but also the generated ROS-induced damage, results in the alteration of the structure of proteins, including transcription factors, for example, kinases, and phosphatases [29], ultimately leading to alterations of signal transduction pathways [30]. ROS-induced oxidative stress has been linked to abnormal hypermethylation of tumor suppressor genes’ promoter regions and global DNA hypomethylation. On the one hand, the site-specific hypermethylation may result from increased expression of DNMTs or the formation of novel DNMT-containing complexes. On the other hand, the 8-hydroxy-2′-deoxyguanosine (8-OHdG) is an oxidized derivative of 2′-deoxyguanosine and can contribute to DNA hypomethylation by blocking DNA methylation at adjacent cytosine bases. Similarly, another oxidative modification, 5-hydroxymethylcytosine (5hmC), facilitates active DNA demethylation, further promoting hypomethylation. In summary, ROS influences hypermethylation and hypomethylation through distinct mechanisms, relevant to the epigenetic regulation of cell proliferation and malignant transformation, respectively [31].

### 1.4. The Antitumor Effects of Curcumin

Turmeric (*Curcuma longa*) belongs to the ginger (Zingiberaceae) family and is native to Southeast Asian countries [32]. Turmeric, renowned for its taste and color, is a frequently utilized spice in crafting curries across India and other Asian nations. Apart from its role as a natural food colorant, for centuries, curcumin has been a cornerstone of holistic healing systems such as Ayurveda and Traditional Chinese Medicine, used for respiratory infections, wound healing, and dermatological ailments [33,34,35].

Active ingredients of turmeric are mostly curcuminoids that are natural polyphenols, such as curcumin, also known as diferuloylmethane (1,7-bis(4-hydroxy-3-methoxyphenyl)-1,6-heptadiene-3,5-dione), dihydrocurcumin, demethoxycurcumin, and bisdemethoxycurcumin [34]. Studies conducted on curcumin elucidated its antioxidant, anti-inflammatory [36], antimicrobial [37,38], antineoplastic [39,40] and anticancer properties. For example, curcumin exerted antitumor effects in skin cancer cell line SRB12-p9 and in vivo in a skin cancer model [41]. Furthermore, curcumin in SKH-1 mice ameliorated the UV-B radiation-induced skin cancer formation [42].

Curcumin’s anticancer effects stem from its pro-apoptotic, anti-angiogenic, and immunomodulatory capabilities, which result from reactivating tumor suppressor genes [43].

Curcumin in a lower concentration as an antioxidant ameliorates UV-B-induced damage in HaCaT cells [44]. However, in a high concentration (at least 20 μM) in human gastric cancer cells (hGCCs), it is a prooxidant that can induce nuclear DNA damage in a dose-dependent manner, activating P53-dependent signal transduction that ultimately leads to apoptosis and downregulation of *DNMT1* expression; an interesting contradiction is that both ROS and curcumin in high doses induce oxidative damage in a dose-dependent manner [45].

Curcumin in 15 µM or higher concentration exerts cytotoxic effects in cancer cells [46]. According to Goel et al., curcumin sensitizes cancer cells to chemotherapeutics by downregulating antiapoptotic proteins such as STAT3, NF-κB, COX2, and Akt, of growth factor receptors, as well as multidrug-resistance proteins [47]. On the other hand, for normal cells, curcumin is cytoprotective, e.g., by activating NRF2, induction of antioxidant enzymes, etc. [47].

Curcumin and similar compounds with diarylheptanoids structure manifest great medicinal potential, e.g., anticancer agents [48]. Curcumin and its major metabolite tetrahydrocurcumin, have a potential covalent blocking and catalytic thiol group inhibition of DNMT1 [49]. Furthermore, curcumin is capable of inducing in vitro a 15–20% decrease in global DNA methylation level [50]. Furthermore, curcumin can modify histone acetylation and deacetylation, exerting anticancer effects by inhibiting DNMTs and HDACs, leading to altered gene expression relevant to cancer development. Curcumin pretreatment in UV-B-exposed mice ameliorated the induced inflammation. The link between curcumin-induced DNA demethylation and apoptosis in cancer cells requires further investigation [49,50,51].

### 1.5. Aims and Scope

In this article, we aimed to explore the potential protective effect of curcumin in high doses against UV radiation-induced *DNMT1*, *DNMT3A*, *DNMT3B*, *HDAC5*, and *HDAC6* expression levels in immortalized keratinocytes (HaCaT), hepatocellular carcinoma (HepG2), and lung adenocarcinoma (A549) cells. Mapping epigenetic alterations caused by UV-B-induced DNA damage and ROS compared against chemopreventive effects of curcumin may elucidate carcinogenesis and progression mechanisms and ultimately promote the development of novel biomarkers to advance cancer diagnostics and potentially improve supplementary therapeutic strategies.

## 2. Results

### 2.1. HaCaT Cell Line

**Figure 1:**
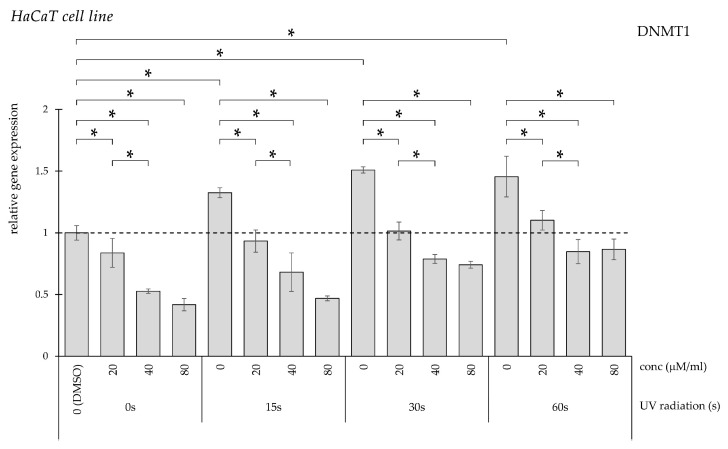
Relative *DNMT1* gene expression in HaCaT cells (* *p* < 0.05) without UV irradiation and after UV irradiation for 15, 30, and 60 s, followed by treatment with DMSO (control) or curcumin at concentrations of 20, 40, or 80 μM.

UV irradiation for 15, 30, and 60 s significantly increased DNMT1 gene expression compared with non-irradiated controls (Figure 1). Treatment with curcumin at concentrations of 20, 40, and 80 μM/mL attenuated the UV-induced elevation in a concentration-dependent manner.

**Figure 2:**
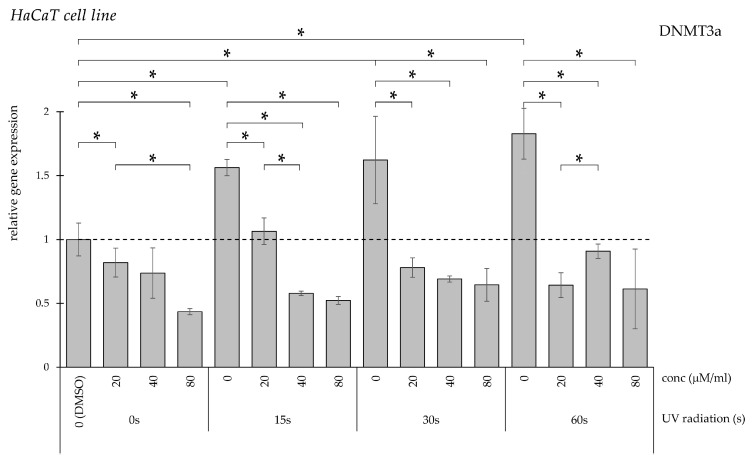
Relative *DNMT3a* gene expression in HaCaT cells (* *p* < 0.05) without UV irradiation and after UV irradiation for 15, 30, and 60 s, followed by treatment with DMSO (control) or curcumin at concentrations of 20, 40, or 80 μM.

UV exposure similarly enhanced DNMT3a transcriptional activity across all examined durations (Figure 2). Administration of curcumin significantly reduced the increased expression observed in irradiated groups at all tested concentrations.

**Figure 3:**
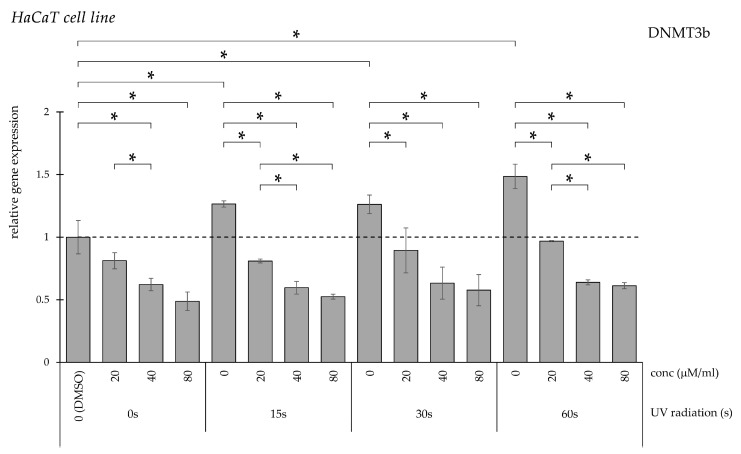
Relative *DNMT3b* gene expression in HaCaT cells (* *p* < 0.05) without UV irradiation and after UV irradiation for 15, 30, and 60 s, followed by treatment with DMSO (control) or curcumin at concentrations of 20, 40, or 80 μM.

DNMT3b expression was also significantly elevated following UV treatment regardless of exposure duration (Figure 3). Curcumin treatment resulted in a dose-dependent decrease in DNMT3b levels compared with UV-treated controls.

**Figure 4:**
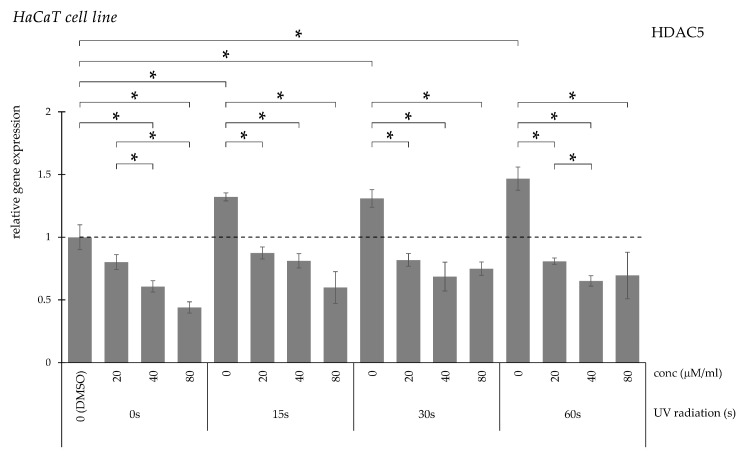
Relative *HDAC5* gene expression in HaCaT cells (* *p* < 0.05) without UV irradiation and after UV irradiation for 15, 30, and 60 s, followed by treatment with DMSO (control) or curcumin at concentrations of 20, 40, or 80 μM.

HDAC5 gene expression increased significantly after UV irradiation (Figure 4). This elevation was significantly suppressed following curcumin administration at 20, 40, and 80 μM/mL.

**Figure 5:**
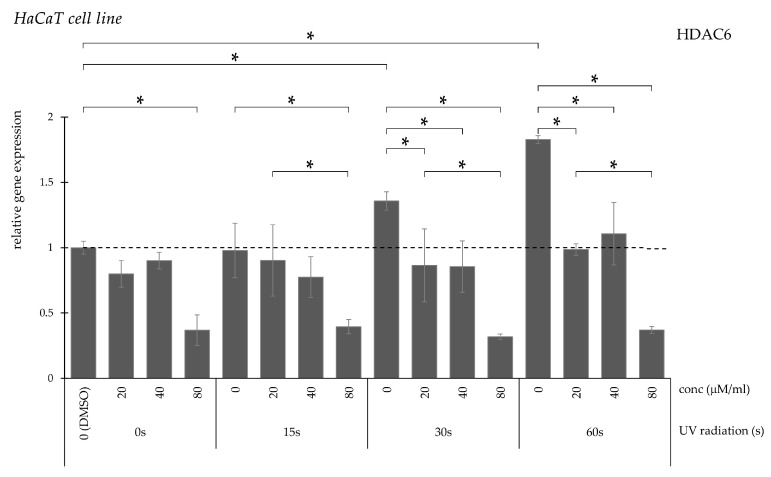
Relative *HDAC6* gene expression in HaCaT cells (* *p* < 0.05) without UV irradiation and after UV irradiation for 15, 30, and 60 s, followed by treatment with DMSO (control) or curcumin at concentrations of 20, 40, or 80 μM.

HDAC6 transcription demonstrated a duration-dependent increase after UV exposure (Figure 5). In the 15 s exposure group, only the highest curcumin concentration reversed the elevation, whereas in the 30 and 60 s groups all tested concentrations significantly reduced expression.

### 2.2. HepG2 Cell Line

**Figure 6:**
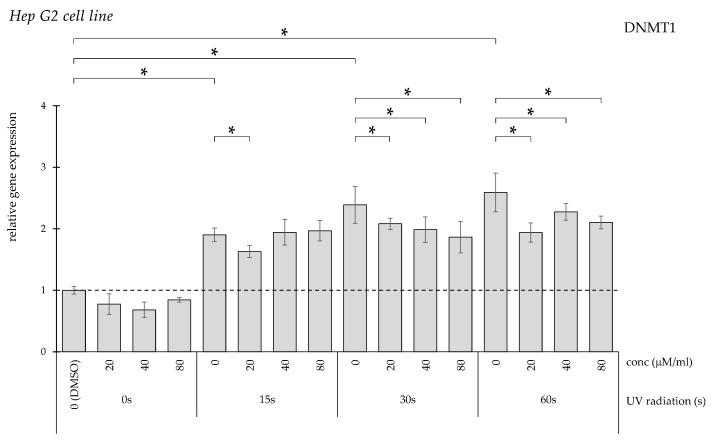
Relative *DNMT1* gene expression in HepG2 cells (* *p* < 0.05) without UV irradiation and after UV irradiation for 15, 30, and 60 s, followed by treatment with DMSO (control) or curcumin at concentrations of 20, 40, or 80 μM.

UV exposure significantly increased DNMT1 expression in a duration-dependent manner (Figure 6). In the 15 s exposure group, reduction was observed only following treatment with 20 μM/mL curcumin, while all concentrations significantly decreased expression after longer irradiation periods.

**Figure 7:**
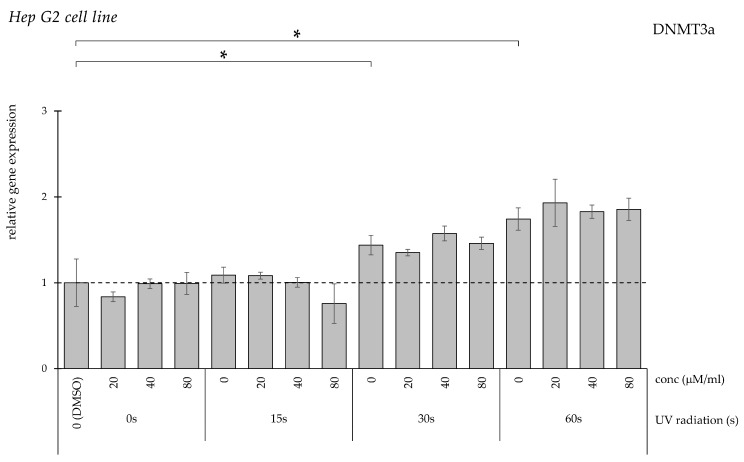
Relative *DNMT3a* gene expression in HepG2 cells (* *p* < 0.05) without UV irradiation and after UV irradiation for 15, 30, and 60 s, followed by treatment with DMSO (control) or curcumin at concentrations of 20, 40, or 80 μM.

DNMT3a expression increased significantly only following 30 and 60 s UV exposure (Figure 7). Curcumin treatment did not significantly modify this elevation.

**Figure 8:**
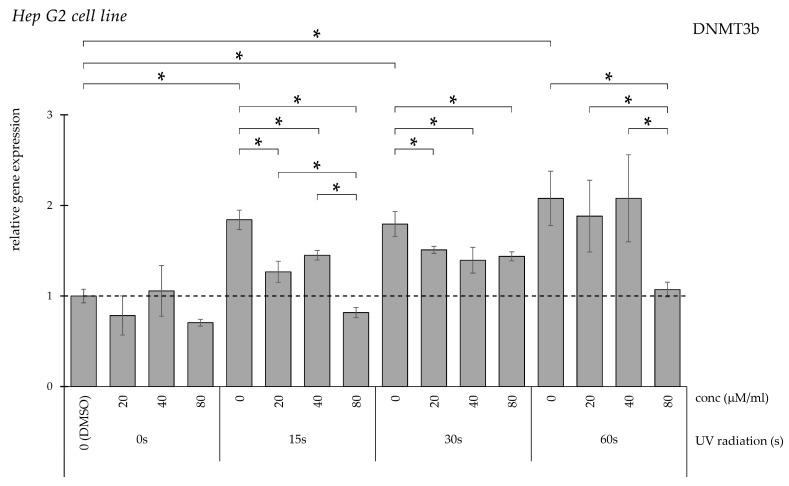
Relative *DNMT3b* gene expression in Hep G2 cells (* *p* < 0.05) without UV irradiation and after UV irradiation for 15, 30, and 60 s, followed by treatment with DMSO (control) or curcumin at concentrations of 20, 40, or 80 μM.

The UV radiation for 15, 30, and 60 s significantly elevated *DNMT3b* expression when compared to the non-irradiated controls (Figure 8). A total of 20, 40, and 80 µM/mL curcumin solution significantly reduced the elevated *DNMT3b* expression in 15 and 30 s UV-treated groups. In the 60 s UV-treated group, the elevation in *DNMT3b* expression was reversed with the highest concentration of curcumin, 80 µM/mL.

**Figure 9:**
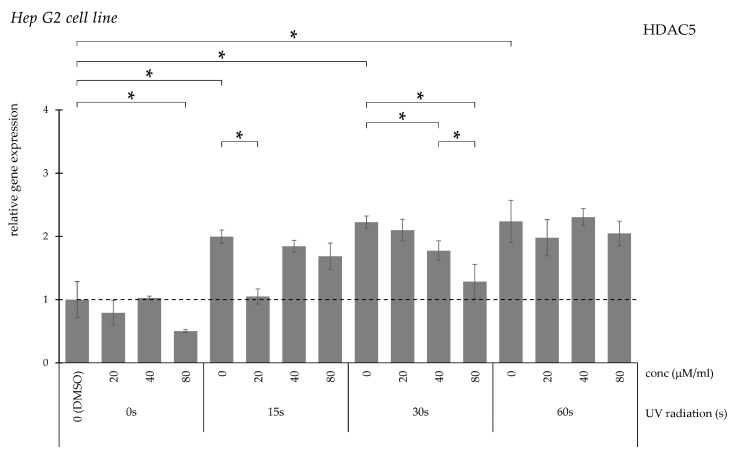
Relative *HDAC5* gene expression in Hep G2 cells (* *p* < 0.05) without UV irradiation and after UV irradiation for 15, 30, and 60 s, followed by treatment with DMSO (control) or curcumin at concentrations of 20, 40, or 80 μM.

HDAC5 expression increased with prolonged UV exposure (Figure 9). Reduction was achieved with lower curcumin concentration following short exposure, while higher concentrations were required after longer irradiation. No significant reduction was observed after 60 s.

**Figure 10:**
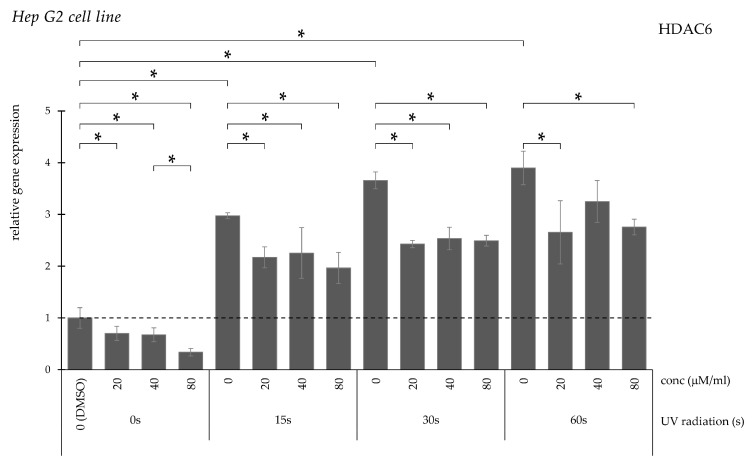
Relative *HDAC6* gene expression in Hep G2 cells (* *p* < 0.05) without UV irradiation and after UV irradiation for 15, 30, and 60 s, followed by treatment with DMSO (control) or curcumin at concentrations of 20, 40, or 80 μM.

HDAC6 expression increased proportionally with irradiation duration (Figure 10). Curcumin significantly decreased expression at all concentrations following 15 and 30 s exposure. After 60 s, reductions occurred only at 20 and 80 μM/mL.

### 2.3. A549 Cell Line

**Figure 11:**
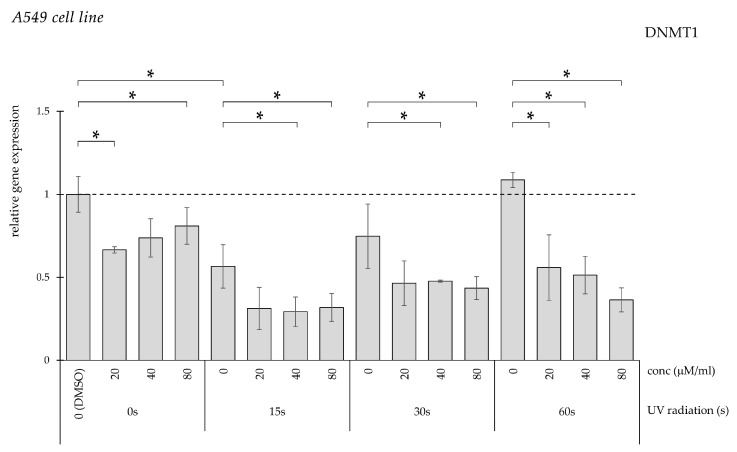
Relative *DNMT1* gene expression in A549 cells (* *p* < 0.05) without UV irradiation and after UV irradiation for 15, 30, and 60 s, followed by treatment with DMSO (control) or curcumin at concentrations of 20, 40, or 80 μM.

DMSO-treated controls demonstrated significantly elevated DNMT1 expression compared with untreated cells (Figure 11). Curcumin treatment significantly reduced expression at all tested concentrations, showing a comparable effect in UV-exposed groups.

**Figure 12:**
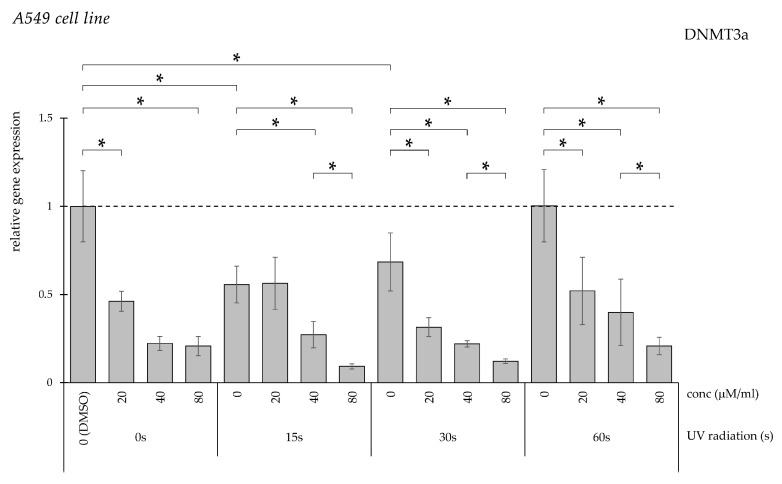
Relative *DNMT3a* gene expression in A549 cells (* *p* < 0.05) without UV irradiation and after UV irradiation for 15, 30, and 60 s, followed by treatment with DMSO (control) or curcumin at concentrations of 20, 40, or 80 μM.

When we measured the expression of *DNMT3a*, the DMSO-treated control group also had a significantly higher level, which was, in turn, reduced with the addition of curcumin solution (Figure 12). In the UV light-treated groups, curcumin could significantly decrease the *DNMT3a* expression in a dose-dependent manner.

**Figure 13:**
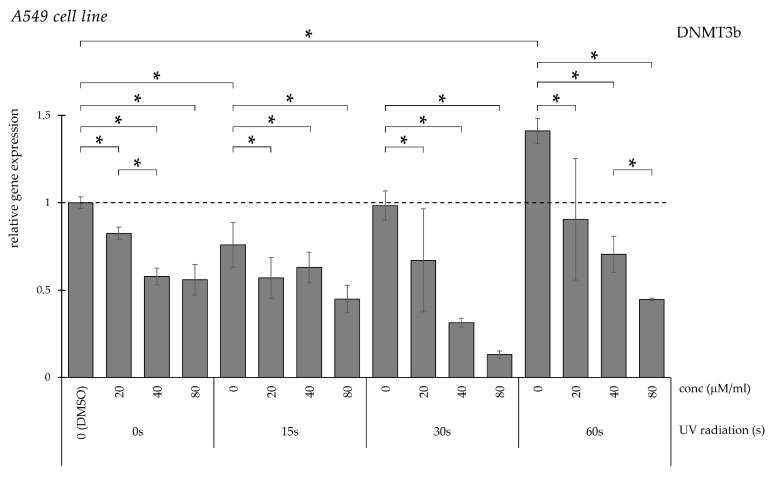
Relative *DNMT3b* gene expression in A549 cells (* *p* < 0.05) without UV irradiation and after UV irradiation for 15, 30, and 60 s, followed by treatment with DMSO (control) or curcumin at concentrations of 20, 40, or 80 μM.

The longest UV exposure significantly increased DNMT3b expression (Figure 13). Curcumin treatment reversed this increase in a dose-dependent manner across irradiation conditions.

**Figure 14:**
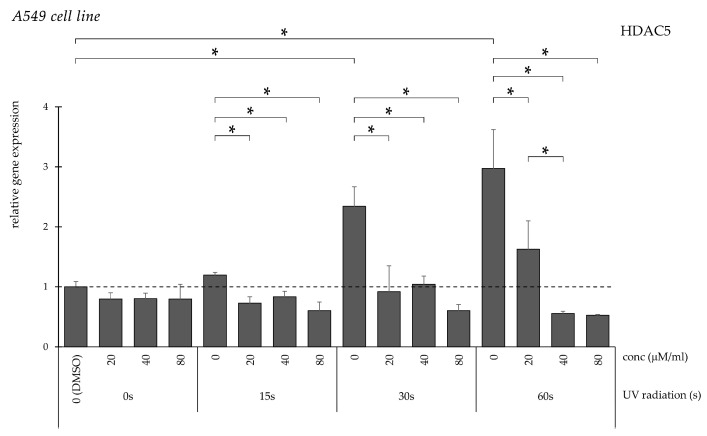
Relative *HDAC5* gene expression in A549 cells (* *p* < 0.05) without UV irradiation and after UV irradiation for 15, 30, and 60 s, followed by treatment with DMSO (control) or curcumin at concentrations of 20, 40, or 80 μM.

HDAC5 expression remained unchanged following short UV exposure but increased significantly after longer irradiation (Figure 14). Curcumin significantly reduced expression in these groups, with the strongest effect observed at 80 μM/mL.

**Figure 15:**
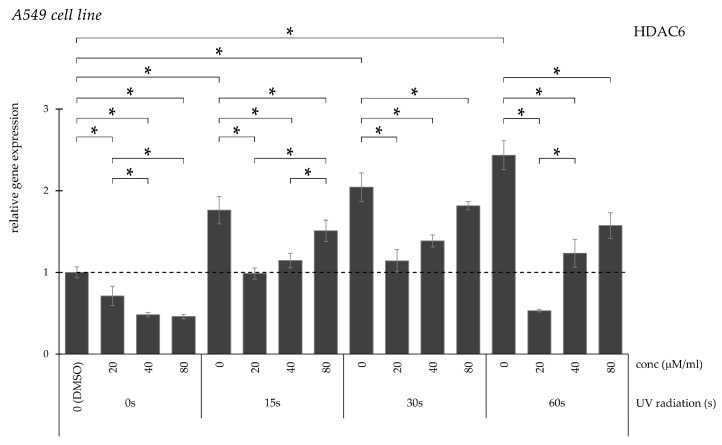
Relative *HDAC6* gene expression in A549 cells (* *p* < 0.05) without UV irradiation and after UV irradiation for 15, 30, and 60 s, followed by treatment with DMSO (control) or curcumin at concentrations of 20, 40, or 80 μM.

Curcumin treatment significantly reduced HDAC6 expression in DMSO-treated controls (Figure 15). UV irradiation induced a progressive increase proportional to exposure duration, which was significantly attenuated by curcumin in a concentration-dependent manner.

## 3. Discussion

The three cell lines show different gene expression patterns after the UV and curcumin treatment. This may be because the affected cell can determine the impact of ROS because of the antioxidant defense capacity of the affected cells [52]. When ROS generation exceeds the capacity of antioxidant defense mechanisms, it can lead to cellular damage (e.g., DNA fragmentation and lipid peroxidation) that diverges to apoptosis or mutation [52,53].

ROS can influence DNMT and HDAC expression and their activity in multifarious ways. Due to several mechanisms, ultimately, it inhibits DNMT and causes global DNA hypomethylation while hypermethylating tumor suppressor genes [54]. Furthermore, ROS can activate cellular inflammatory signal transduction mechanisms that increase DNMT activity, ultimately increasing the likelihood of malignant transformation [55].

### 3.1. The Effects of UV Exposure on the Examined DNMTs and HDACs

Generally, UV exposure increased the DNMT and HDAC gene expression levels in the examined cells dose-dependently. This is explained by the fact that UV light can affect the catalase enzyme and upregulate nitric oxide synthase (NOS) synthesis, which ultimately induces ROS generation [52] that can induce DNMT and HDAC enzyme expression pattern alterations. UV exposure may also decrease protein kinase C (PKC) expression, thereby increasing ROS production further [52]. Contrary to our results, the expression of *HDACs* was reduced by UV exposure in fibroblast cells according to Lee et al. [56].

DNA-damage-induced expression of DNMT enzymes is explained by the fact that DNMT and HDAC enzymes are involved in DNA damage repair in multifarious ways [57,58,59]. DNMT1 contributes to DNA mismatch repair, interacting with DNA repair proteins through mediators such as methyl-CpG binding domain 4 (MBD4) and proliferating cell nuclear antigen (PCNA). Furthermore, DNMT1 binds to the CHK1 protein, thereby activating P53, which regulates cell cycle arrest and apoptosis [60]. DNMT3A can interact with P53 directly repressing P53-mediated transactivation of the *P21* tumor suppressor gene, which takes part in cell-cycle arrest [61,62]. On the other hand, DNMT3B is responsible for the integrity of the centromere region by restricting R-loop-mediated DNA damage [63].

HAT and HDAC enzymes acetylate/deacetylate lysine not only on histones but also modulate P53 protein, among others, taking part in DNA damage response [64]. Hence, acetylation of lysine 120 (K120) of P53 is a response to DNA damage and activates the transcription of *bcl-2-like protein 4* (*BAX*) and *P53 upregulated modulator of apoptosis* (*PUMA*) proapoptotic genes [65]. However, HDAC5 suppresses K120 acetylation, regulating P53 toward activating P21 and antioxidant genes during the early stages of stress. Furthermore, genotoxic stress in vivo downregulates *HDAC5* expression, which determines P53-mediated cell fate toward apoptosis [66].

HDAC6 deacetylates MutL homolog 1 (MLH1) and thereby disrupts MutSα–MutLα complex, which is a DNA damage sensor. Thus, HDAC6 activity increases the tolerance of DNA damage [67]. Nuclear-localized HDAC6 binds to and deacetylates ten-eleven translocation 2 (TET2), triggering active DNA demethylation and leading to DNA damage formation through thymine DNA glycosylase-mediated excision mechanism [68].

The examined gene effects seem to balance each other. The interactions of DNMT and HDAC enzymes, among others, orchestrate a balance in the regulation of gene expression, for example, *P53*, involved in DNA damage repair and apoptosis. It has been hypothesized earlier that DNA damage-induced DNMT enzyme expression does not cause a genome-wide DNA hypermethylation [69].

ROS depletes important antioxidant molecules such as glutathione (GSH), S-adenosylmethionine (SAM), and S-adenosylhomocysteine (SAH), leading to global DNA hypomethylation [70,71]. Decreased SAH levels specifically activate the DNMT1 enzyme, resulting in the hypermethylation of CpG regions in tumor suppressor genes such as P53 and phosphatase and tensin homolog (PTEN) gene [72,73]. The downregulation of PTEN upregulates PI3K/Akt/mTOR and NF-κB signaling pathways [73], which can lead to the aforementioned DNMT1 activation [74]. This activation could have theoretically led to a negative feedback mechanism. Indeed, the UV-B-generated DNA damage and ROS can activate proinflammatory signal transduction, for example, NF-κB, and can theoretically increase *DNMT* expression, but NF-κB can also paradoxically downregulate *DNMT1* expression in a redox-sensitive manner [55,75,76].

UV exposure and ROS induce TGFβ signal transducer, which is a growth inhibitor for keratinocytes and ovarian cells [77,78], and it increases the expression and activity of DNMT1, DNMT3A, and DNMT3B [79]. Furthermore, ROS activates PKC [80]. For example, in vitro phosphorylation of DNMT1 by PKCζ reduces its methyltransferase activity. Conversely, PKC’s phosphorylation of human DNMT1 is isoform-specific [81]. ROS also typically inhibits HDAC enzymes but induces their gene expression, potentially with a negative feedback loop regulation [54]. ROS (for example, hydroxyl groups (OH·) and superoxides (O_2_·^−^)) can also oxidize thiol groups derived mostly from cysteine [29]. Thus, the oxidative stress of ROS in vitro decreased the GSH level and induced the expression of *ten-eleven translocation1* (*TET1*) gene, which is a methyl-cytosine dioxygenase enzyme taking part in DNA demethylation [82,83]. Apart from that, curcumin induces the gene expression of *TET1*, leading to DNA demethylation, exerting slight effects on global DNA methylation, but it can reactivate tumor suppressor genes in cancer [84,85].

### 3.2. The Effects of Curcumin on the DNMT and HDAC Expression Patterns

The examined gene expression patterns were orchestrated predominantly by UV-B light-induced DNA damage and ROS, combined with curcumin’s prooxidant effects [55]. Curcumin deters cancer cell proliferation and induces apoptosis by modulating DNMT and HDAC activities, which are mostly dose-dependent. Complicated secondary signal transduction mechanisms influenced by redox-sensitive aspects, prooxidant and antioxidant effects, and inflammatory factors orchestrate DNMT and HDAC expressions, which are relevant in regulating cell proliferation and apoptosis. For example, curcumin can reactivate the PTEN, Nrf2, WIF-1, and RARβ2 tumor suppressor genes in cancer. HDAC inhibitors (HDACis) exhibit antitumor effects by activating cell cycle arrest, inducing apoptosis and autophagy, and inhibiting angiogenesis. Curcumin in vitro inhibits UV-B-induced *MMP-1* and *MMP-3* expression, ROS generation, MAPK/NF-κB/AP-1, phosphorylation of P38, and c-Jun N-terminal kinase [45,86,87].

The curcumin forms weak covalent bonds, the so-called Michael adducts, with thiol groups, including GSH, as well as the catalytic site of DNMT and HDAC enzymes, which is crucial for their activity and influences their gene expression through feedback mechanisms [88]. The GSH content of cells can quench ROS and influence redox-dependent signal transducers by altering charges on the surface of macromolecules, resulting in altered gene expression patterns. Curcumin ultimately induces apoptosis by inhibiting DNMT1 elevated by mTOR in all examined cell lines, while it upregulates lncRNA NBR2, which is an inhibitor of the Akt/mTOR [74,89,90].

In mice psoriatic inflammation model, increased DNMT1 expression and DNA methylation were associated with increased inflammatory signal transducer activity, while the anti-inflammatory mediators were downregulated. However, the balance can be restored with a DNMT1 inhibitor [91]. Curcumin, by suppressing inducible nitric oxide synthase (iNOS) and chemokine receptor type 4 (CXCR-4), can also inhibit other inflammation factors (CRP, inflammatory matrix metalloproteases (MMPs), NF-κB, TNF, LOX, COX2, 5-LOX, IL-1β, IL-6, IL-23, PGE2), carcinogenesis-relevant factors (Activator protein 1 (AP-1), human epidermal growth factor receptor 2 (HER2), epidermal growth factor receptor (EGFR)), and STAT3 phosphorylation, *BCL2*, cyclin D1, etc. [92]. Despite the different expression ratios, the patterns of the examined genes were nearly similar. The underlying cause of the phenomenon is that curcumin, without UV exposure, decreased the examined gene expressions in a dose-dependent manner in the HaCaT cell line. The anti-inflammatory effects of curcumin inhibit *DNMT* expression, contributing to the autoinhibitory feature of DNMT1 enzyme [91]. Furthermore, the structure and effects of DNMT3A and DNMT3B share some similarities, and DNMT enzymes are linked to HDAC enzymes, orchestrating epigenetic regulation of cell maintenance and apoptosis, cross-regulating each other [93].

Curcumin treatment in HaCaT cells activates the apoptosis-inducing factor (AIF) and caspase 3 [94,95]. Indeed, HDACi effects combined with caspase 3 activation in vitro promoted apoptosis, which supports the anticancer effect of curcumin [96]. Another protective mechanism of curcumin is explained via the nuclear factor erythroid 2-related factor 2 (Nrf2) [97]. The complex of Nrf2 and its inhibitor, Keap1, is located in the cytoplasm until an electrophilic compound interacts. Upon the stimulus from an electrophile molecule, the linkage between Nrf2 and Keap1 breaks, leading to increased antioxidant production [98]. Natural polyphenols, including curcumin, can stimulate the fragmentation of Nrf2 and Keap1 [99]. The Nrf2 signaling pathway is present in all cells and crucial in ROS neutralization, cytoprotection, drug and endobiotics metabolism, and tumor prevention [100]. For example, in HaCaT cells, curcumin ameliorated UV exposure-induced photo-damage by activating Nrf2 expression [101].

In HepG2 cells, ROS exposure was mostly quenched by GSH, and without UV-B exposure, curcumin only decreased the HDAC5 and HDAC6 expression [102]. In hepatocytes, GSH concentration can reach approximately 10 mM, while in most cells, it ranges from 1 to 2 mM [76]. In these cells, UV exposure caused DNA damage, and the consequent ROS exposure was mostly quenched by GSH. GSH content also influences the redox-sensitive nucleotide excision repair (NER) and the connected DNMT enzyme activity [103,104]. GSH, as a substrate of glutathione transferase (GST) enzymes takes part in regulating cell proliferation, too, while blocking GST can be a potential anticancer approach in this context [105]. Hence, curcumin can spontaneously form Michael adducts with GSH, acting as a substrate inhibitor of glutathione *S*-transferase (GST). Furthermore, curcumin may form a Michael adduct with cysteine near the active site of GSTP1-1, causing enzyme inhibition [106]. Even paradoxically increasing oxidative stress was accompanied by GSH level increasing, while significantly elevated *DNMT1* and *DNMT3b* expressions were observed [107]. This may contribute to curcumin’s prooxidant effect, increasing some examined *DNMT* and *HDAC* expressions. The depletion of GSH in HepG2 cells exacerbates cancer [108], while increased GSH levels exerted cytoprotective effects [109].

On the other hand, the prooxidant effects of curcumin are quenched by the GSH, but apart from that, the HDACi effects of curcumin in combination with the UV-B-generated ROS could still activate apoptosis-inducing factors and, with a negative feedback regulation, increase the examined DNMT and HDAC levels [54,110].

The cellular GSH level in A549 cells is elevated seven-fold higher compared to the normal human lung fibroblast line (CCL-210). Still, the GSH content could quench the DMSO-induced overexpression of examined genes, but without UV-B exposure, the *DNMT1* expression could be similarly induced by curcumin with negative feedback regulation. The interaction of GSH with curcumin can be responsible for altering the GSH synthetic cycle, ultimately influencing the redox balance of important signal transducer proteins such NF-κB [55,75,76]. GSH content influences the redox-sensitive NER and the connected DNMT enzyme activity, too [103,104]. UV-B exposure increased the examined gene’s expression as expected, but curcumin treatment decreased it in certain experimental setups in a dose-dependent manner, except for *HDAC6* expression, which can be explained by catalase activity. The impact of UV-B exposure on catalase enzyme activity was found to be both pH-sensitive and dependent on oxygen levels and the cell’s internal redox state, respectively. These factors determine whether catalase activity under UV exposure results in protective effects or cytotoxicity [111]. Due to the different ratio of oxidized glutathione (GSSG)/basic reduced glutathione (GSH) in HepG2 and A549 cells, the inhibition of GSH by curcumin could support prooxidant mechanisms in A549 cells, increasing *HDAC6* expression to increase DNA damage response [64,106].

Curcumin in AML cells downregulates DNMT1 expression by decreasing the expression of P65 and SP1 positive regulators, leading to P15(INK4B) reactivation by hypomethylation of its promoter [26]. On a molecular and cellular level, curcumin can inhibit epithelial-to-mesenchymal transition and influence various targets implicated in melanoma initiation and progression, such as BCl2, MAPKs, p21, and specific microRNAs [43]. miRNAs modulated by curcumin (miR-15a, miR-16, miR-21, miR-22, miR-26, miR-101, miR-146, miR-200, miR-203, and let-7) restore the epigenetic regulation balance between HAT and (HDAC1, HDAC3, HDAC4, HDAC5, HDAC8) activating or inactivating the expression of genes relevant in cancer [112].

Curcumin downregulates immunoregulatory signaling pathways that may encourage persistent inflammation and an antiapoptotic state [113]. Examples are the STAT3 protein and NF-κB transcription factor, which support cell survival, proliferation, angiogenesis, immune evasion, and tumor resistance and can be dysregulated in several chronic inflammatory diseases and different types of cancers [114,115]. The STAT proteins are part of transcription factors found in the cytoplasm, and they play significant roles in regulating cell survival, cellular proliferation, and development [116]. STAT1 and STAT3 have opposing effects in malignant diseases; STAT1 exhibits anti-oncogenic effects, whereas STAT3 supports tumor progression [117]. The transcription factor NF-κB transmits cellular signals and regulates cytokine synthesis, making it crucial during immune responses and cellular development [118]. The activity of NF-κB is directly regulated by the phosphorylation of its inhibitor, IκB, in the cytoplasm [119]. The NF-κB pathway is complex and demonstrates varying effects, either anti- or pro-inflammatory, as well as tumor-suppressing or -promoting, depending on the activation factors [115]. Curcumin exhibits anti-inflammatory and antineoplastic effects by interrupting the IκB phosphorylation and translocation of NF-κB into the nucleus and suppressing STAT3 protein and its mRNAs [113].

Furthermore, curcumin increases gene expression and activates peroxisome proliferator-activated receptor gamma (PPARγ), suppressing NF-κB expressions and activities too [120,121]. Besides NF-κB, the NOTCH and BCL2 signaling are downregulated by curcumin treatment [122].

In summary, curcumin can partly protect both DNMT and HDAC enzymes from ROS-induced damage. DNMT1 possesses an AP-1 regulatory region in P19 cells but not Y1 cells that react to cellular DNA methylation capacity, resulting in negative feedback regulation of DNMT1 expression [123]. This may explain the negative feedback regulation of DNMT1 expression.

### 3.3. The Effects of DMSO on the Examined DNMTs and HDACs

In HaCaT and A549 cells in the DMSO-treated control groups, the DNMT and HDAC gene expression levels were also increased compared to the untreated control groups. However, in HepG2 cells, DMSO could not significantly increase the mentioned parameters in the same experimental setup. The pleiotropic effects of DMSO can explain this, which can modify differently the expression pattern of *DNMT1*, *DNMT3a*, and *DNMTb* genes [124]. Furthermore, DMSO generally exerts antioxidant effects but can behave as a prooxidant, particularly at doses ≥1%, which induces oxidative stress. For example, paradoxically, it exacerbates the oxidation of thiol groups of proteins caused by hydrogen peroxide (H_2_O_2_) in vitro. Oxidative stress can decrease the activity of DNMT and HDAC enzymes in HaCaT and A549 cells [86,125]. The inactivation of DNMT and HDAC enzymes consequently increases their gene expression through a negative feedback loop to maintain balance [126]. This explains how DMSO significantly induced the examined gene’s expression compared to the untreated control in the HaCaT and A549 cells.

In HepG2 cells, DMSO could not significantly alter the expression levels of the examined genes compared to the untreated control. ROS can activate NF-κB, while DMSO can inhibit NF-κB in a redox-sensitive manner, leading to wide-branching molecular biological effects, for example, gene expression [76]. Activation of NF-κB by TNF and IL-6 can induce DNMT1 recruitment to chromatin, suppressing breast cancer metastasis suppressor 1 (BRMS1) gene [74]. The high GSH content can quench the DMSO-generated ROS, thereby mitigating DNA damage, decreasing the active-driven inflammatory signal transduction, and ultimately not changing the *DNMT* and *HDAC* expression [127].

### 3.4. Limitations

This study has certain limitations. Gene expression was assessed at the mRNA level; therefore, corresponding changes in protein levels and enzymatic activity of DNMTs and HDACs were not directly examined. In addition, the experiments were performed in established cell lines under in vitro conditions, which may not fully reflect the complexity of in vivo biological systems. For example, theoretically non-specific stress or prooxidant effects at high curcumin concentrations could influence protein functions (apart from the mentioned Michael-reaction) or interfere with substrates (e.g., GSH), etc. Thus, further protein-level and in vivo studies are warranted to confirm and extend these findings. Therefore, the supposed synergistic effects with other anticancer therapies need to be underpinned experimentally in the future.

DMSO itself is capable of altering DNMT and HDAC expression in some cell lines. Although DMSO is a widely accepted solvent, all curcumin treatments were performed in the presence of 1% DMSO; thus, to distinguish the effects of curcumin from those of the solvent or their interaction can be performed only in a relativistic context.

The expression level of the examined genes does not represent the whole epigenetic regulation and protein-level feature of the examined cells, including enzyme activities, and the level of substrates, antioxidants, etc. Thus, the result that utilized high doses of curcumin treatment restored the UV-induced examined gene expression level changes in some cell lines does not refer to non-coding RNA level, or UV-induced gene mutations, etc. To elucidate the effects of high doses of curcumin on all other factors connected to UV exposure requires specific studies, out of the scope of this article. For example, both mRNA expression and protein content and functions of *NF-κB*, *Nrf2*, *TET1*, *PKC* genes, and miR-15a, miR-16, miR-21, miR-22, miR-26, miR-101, miR-146, miR-200, miR-203, and let-7 miRNAs levels, GSH level, etc., corresponding to UV exposure and curcumin treatment are warranted to be mapped in the future.

The bioavailability of curcumin is poor, predominantly due to the quick metabolic elimination caused mostly by the uridine-5′-diphosphoglucuronosyltransferase (UGT) enzymes, especially the hepatic UGT1A1 enzyme and the intestinal UGT1A8 and UGT1A10 [128]. This problem could be theoretically solved by the consumption of piperine, which inhibits UGT enzymes [129]. However, the inhibition of UGT enzymes can influence some metabolic diseases as well as the metabolism of some active pharmaceutical ingredients, requiring medical attention.

## 4. Materials and Methods

### 4.1. Cell Culture

The HepG2, A549, and HaCaT cell (CLS Cell Lines Service GmbH, Eppelheim, Germany) were grown in Dulbecco’s Modified Eagle Medium (DMEM) (Sigma Aldrich, Schnelldorf, Germany), which was supplemented with 10% fetal bovine serum, 100 U/mL penicillin, and 0.1 mg/mL streptomycin. They were kept in an incubator set at 37 °C and 5% CO_2_. Once the cells had reached 80% confluency, they were detached by using Trypsin-EDTA (Sigma Aldrich, Schnelldorf, Germany) and then seeded in 6-well culture plates for further treatment.

### 4.2. Treatment

HaCaT, HepG2, and A549 cells were exposed to UV radiation and curcumin under the following conditions: the cells were then exposed to UV-B radiation at 125 mJ/cm^2^ using a Bio-Link BLX-312 UV-B lamp (Vilber Lourmat GmbH, Marne-la-Vallée, France) for different time intervals (15, 30, and 60 s). The cells were treated with different concentrations of curcumin solution: 20 µM, 40 µM, and 80 µM for a total of 24 h immediately given after UV-B exposure. Curcumin was dissolved in DMSO (Sigma Aldrich, Schnelldorf, Germany), and in all experimental setups, the concentration of DMSO was adjusted to 1%. In 0 µM curcumin control groups, only DMSO was given.

### 4.3. RNA Extraction

Total RNA was isolated from treated and control samples using ExtraZol Tri-reagent (Nucleotest Bio Kft., Budapest, Hungary) according to the manufacturer’s acid guanidinium thiocyanate–phenol–chloroform extraction protocol. Cells were lysed directly in reagent, followed by chloroform-mediated phase separation. RNA was precipitated using isopropanol, washed with 75% ethanol, briefly air-dried, and resuspended in nuclease-free DEPC-treated water. RNA purity and concentration were verified spectrophotometrically (A260/A280 ≈ 2.0). Samples were stored at −80 °C until analysis.

### 4.4. qRT-PCR

qRT-PCR reactions were prepared after complete thawing of all components. To prevent premature reverse transcription, the KAPA RT Mix (Sigma Aldrich, Schnelldorf, Germany) and assembled reactions were maintained on ice during preparation. Primers were diluted to working concentrations using PCR-grade water (Sigma Aldrich, Schnelldorf, Germany). Each reaction contained 10 μL KAPA Master Mix, 0.4 μL KAPA RT Mix, 0.4 μL forward primer, 0.4 μL reverse primer, 1 μL mRNA template, and PCR-grade water to a final volume of 20 μL.

Reverse transcription was performed at 42 °C for 5 min followed by polymerase activation at 95 °C for 5 min. Amplification consisted of 45 cycles at 95 °C for 5 s, 56 °C for 15 s, and 72 °C for 5 s.

Melting curve analysis (95 °C for 5 s, 65 °C for 60 s, and continuous heating to 97 °C) confirmed amplification specificity and amplicon purity.

HPRT1 served as the internal reference gene. Primer sequences for HPRT1 and DNMT1, DNMT3A, DNMT3B, HDAC5, and HDAC6 are presented in Table 1.

The expression levels of *DNMT1*, *DNMT3A*, *DNMT3B*, *HDAC5*, and *HDAC6* were evaluated and compared to the expression level of the *HPRT1* gene.

### 4.5. Statistical Analysis

The Shapiro–Wilk (SW) test was used to examine the normality of the qRT-PCR data. Based on the distribution of the data, one-way ANOVA and Tukey’s post hoc test were used to analyze the qRT-PCR results. Results were considered statistically significant if the *p*-value was less than 0.05.

## 5. Conclusions

Although the inhibitory effects of curcumin on DNMT and HDAC enzymes have been known previously, the gene expression level of a specific gene panel was analyzed, and the comparison across multiple cell lines under UV-B exposure demonstrated in this study.

In our experimental setup, curcumin restored the altered DNMT and HDAC patterns after the UV exposure in A549 and HaCaT cells in a dose-dependent manner, presumably by modulating the multifarious secondary signal transduction mechanisms. Hence, the effects of direct DNA damage, ROS, and consequent inflammatory and procarcinogen signal transducers could increase the expression of the examined genes in all the investigated cell lines.

On the other hand, in HepG2 cells, the generally elevated GSH quantity could partly protect DNA from direct damage and partly ameliorate the effects of ROS, leading to a less pronounced, but still significant increase in the examined gene expression levels. This effect was also dose-dependently decreased by curcumin, but in a less marked manner. This refers to the amelioration of the prooxidant effects exerted by both UV exposure as well as the presence of curcumin. Furthermore, the prooxidant effects of curcumin seem to increase DNMT and HDAC methylation, presumably through modulating many signal transduction mechanisms.

In summary, both for cancer prevention and as a supplement to pharmaceutical therapies, curcumin may exert anticancer effects and improve the life expectancy of cancer patients. Both HDAC inhibitors and DNMT inhibitors are promising anticancer approaches. Their combined effects manifest synergy, highlighting the relevance of the restoration of healthy gene expression patterns during cancer treatment. The tissue-specific susceptibility based on gene expression parameters to UV exposure and the chemopreventive effects of curcumin may pave the way toward developing novel supplementary combination therapies. Presumably, combined with other chemopreventive agents through the HDACs and DNMTs activity at the gene expression level, it can exert a synergistic effect. To map those effects, further experiments are warranted.

## Figures and Tables

**Table 1:** Primer sequences of genes of interest: *DNMT1*, *DNMT3A*, *DNMT3B*, *HDAC5*, *HDAC6*, and *HPRT1*.

	Forward	Reverse
***DNMT1***	5′-GGA GCA GGT GGA GAG TTA-3′	5′-GTA GAA TGC CTG ATG GTC TG-3′
***DNMT3a***	5′-GCA GCG TCA CAC AGA AG-3′	5′-GGC GGT AGA ACT CAA AGA AG-3′
***DNMT3b***	5′-GAA CGA CGT GAG GAA CAT C-3′	5′-GGC CTG TAC CCT CAT ACA-3′
***HDAC5***	5′-CAG CAC CAT CGG TTC ATA G-3′	5′-CAG GGA GAG AGT GGG TAA G-3′
***HDAC6***	5′-GCC CAG GCT TCA GTT TC-3′	5′-CCT CGC TCT CCT CTA CAT T-3′
***HPRT1***	5′-TGC TTC TCC TCA GCT TCA-3′	5′-CTC AGG AGG AGG AAG CC-3′

## Data Availability

The original contributions presented in this study are included in the article. Further inquiries can be directed to the corresponding authors.
